# Treatment of blunt splenic injury in children in China

**DOI:** 10.3389/fsurg.2024.1502557

**Published:** 2024-12-03

**Authors:** Wu Wang, Haiyan Lei, Wenhan Zhang, Wenhai Li, Hongqiang Bian, Jun Yang

**Affiliations:** ^1^ Wuhan Children's Hospital (Wuhan Maternal and Child Healthcare Hospital), Tongji Medical College, Huazhong University of Science & Technology, Wuhan, China; ^2^ School of Medicine, Jianghan University, Wuhan, China; ^3^ Clinical Research Center for Pediatric Minimally Invasive Diagnosis and Treatment in Wuhan, Wuhan, China

**Keywords:** paediatric, splenic injury, trauma, non-operative management, hemodynamics

## Abstract

**Introduction:**

Nonoperative management (NOM) is recognized as a viable treatment for pediatric closed splenic trauma. However, clinical guidelines are applied inconsistently, resulting in different treatment strategies in different regions. This study aimed to investigate the independent risk factors influencing the length of stay in pediatric closed splenic injuries and to analyze the key determinants in the choice of surgical treatment to optimize inpatient management and patient care and improve outcomes.

**Methods:**

A retrospective evaluation of medical records of pediatric patients with blunt splenic injury (BSI) admitted to Wuhan Children's Hospital from 2020 to 2024 was conducted. The dataset included demographics, mechanism of injury, injury grade, associated injuries, therapeutic measures, and outcomes, which were subjected to statistical analysis. Factors influencing length of hospital stay and treatment regimen were also analyzed.

**Results:**

A total of 88.5% of patients underwent NOM, with 11% requiring splenic embolization due to hemodynamic instability or arterial hemorrhage. Surgery was required in 11.5% of patients, primarily for combined gastrointestinal perforation, or peritonitis. One patient died due to brain injury. Trauma scores and transfusion requirements were higher in the surgical group (37.7 ± 16.1 vs. 17.2 ± 13.1, *p* < 0.001; 21.7% vs. 100%, *p* < 0.001). Multivariate logistic regression showed that gastrointestinal complications significantly influenced the decision to operate (*p* = 0.0087). A generalized additive model showed a corresponding increase in length of stay with increasing injury severity, with the curve flattening in the mid to high ISS range (40–60).

**Conclusion:**

NOM remains an effective and preferred treatment strategy for pediatric BSI, particularly in the setting of stable hemodynamic parameters. This approach reduces the need for surgical intervention and associated complications while preserving splenic function. The study highlights that gastrointestinal complications are important determinants of surgical management. Further research into long-term outcomes and advancements in conservative management are needed.

## Introduction

Trauma represents the primary cause of mortality and morbidity in children. More than 90% of childhood trauma globally is attributable to blunt force trauma, with approximately 10% of cases involving abdominal and pelvic injuries. Among the abdominal traumas, the spleen is the most frequently injured internal organ in children (1, 2). Children are at a significantly increased risk of damage to abdominal organs compared to adults due to several factors, including thinner abdominal walls, greater force transmission, larger relative surface areas of the spleen and liver, as well as more flexible ribs and a relatively higher position of the diaphragm (3).

The management of splenic trauma has undergone significant changes over the past few decades, particularly in non-operative management (NOM). The scope of non-surgical treatment has gradually expanded from observation and monitoring alone to include angiography and angioembolization (AG/AE), with the central goal of preserving the spleen and its immune function, which is particularly important in pediatric patients. Given the critical role of the spleen in the immune system and the high risk of patients facing compromised immune function after splenectomy, spleen injury may not only be life-threatening at the time of patient admission. Still, it may also result in fatal complications due to the rupture of a delayed subcapsular hematoma or pseudoaneurysm (PSA). Furthermore, post-splenectomy infection (OPSI) represents a significant risk factor for late complications due to the loss of the spleen's immune defenses. The length of hospital stay is an important indicator of the patient's recovery and outcome. In pediatric closed splenic injuries, a prolonged hospital stay may be associated with more severe injuries, the presence of comorbidities, and different treatment options. Therefore, analysis of risk factors for prolonged hospital stays may help to optimize treatment strategies and improve patient prognosis.

Nonoperative management (NOM) has become the standard of care for hemodynamically stable patients with blunt splenic injuries (4). These patients are typically treated with strict bed rest and close monitoring of vital signs. The direct success rate of NOM currently exceeds 98% (5, 6). This study aimed to investigate the independent risk factors influencing the length of stay in pediatric closed splenic injuries and to analyze the key determinants in the choice of surgical treatment to optimize inpatient management and patient care and improve outcomes.

## Methods

### Study design

**Figure 1:**
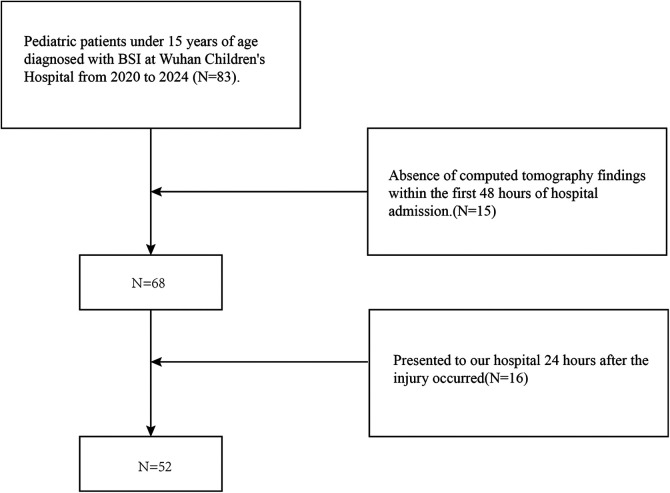
Flow chart of the study participants. BSI, blunt splenic injury.

This was a retrospective single-center cohort study of pediatric patients diagnosed with BSI. The study population comprised patients who were hospitalized or transferred to Wuhan Children's Hospital from January 1, 2020, to May 1, 2024, in Wuhan. The pediatric age group was defined as patients younger than 15 years old, in accordance with the age limit for hospitalization in Chinese pediatric hospitals. There were 83 patients in this group. Patients who were admitted to the hospital after 24 h post-injury (*N* = 16) or who did not have CT findings within 48 h post-injury (*N* = 15) were excluded from the analysis, resulting in a total of 52 patients included in the study (Figure 1).

### Data collection

The data about the patient and the nature of the injury were collated from the hospital's electronic case system, following the requisite approval from the ethical review board. Demographic data, including age, gender, and admission center, were collected. The clinical information obtained included vital signs, transfusion requirements, World Society of Emergency Surgery (WSES) ratings, Injury Severity Score (ISS), hemodynamic status, mechanism of injury, splenic injury grade, and other relevant injuries. Furthermore, the data set includes information on the type of treatment (conservative or surgical), length of stay in the intensive care unit (ICU), total length of stay (LOS), time in bed, and mortality.

The injury severity scores were determined by a team of physicians from the Department of Critical Care Medicine and the Department of Emergency Surgery.

The term “hemodynamic stability” is defined as a systolic blood pressure of 90 mmHg plus twice the child's age in years. It should be noted that the lower limit is below 70 mmHg plus twice the child's age in years, or 50 mmHg in some studies. Furthermore, a positive response to fluid resuscitation was defined as hemodynamic stability. This may be evidenced by a decrease in heart rate, a clear sensation, the return of a peripheral pulse, normal skin color, an increase in blood pressure and urine output, and an increase in extremity warmth (7). As these parameters vary with age, this is determined through a joint clinical judgment between emergency surgeons and pediatric critical care medicine physicians. The initial classification of the grade of injury was made by a pediatric radiologist with expertise in trauma imaging, who evaluated a CT scan within 48 h of the child's admission to the hospital according to the AAST classification system. Nonoperative treatment was defined as bed rest and monitoring of vital signs, including splenic embolization, as it is a less invasive procedure. Surgical treatment was defined as splenectomy or spleen-preserving surgery, which included partial splenectomy and splenectomy. The injury severity scores were determined by a team of physicians from the Department of Critical Care Medicine and the Department of Emergency Surgery. The primary objective of this study was to identify the factors that influence the length of hospitalization and the decision to perform surgery.

### Statistical analysis

All statistical analyses were conducted using the R statistical software package (http://www.R-project.org) and EmpowerStats (http://www.empowerstats.com, X&Y Solutions, Inc., Boston, MA). Descriptive statistics were calculated for each variable. Continuous variables were described as mean ± standard deviation (SD), whereas categorical variables were reported as frequencies and percentages (*n*, %). The appropriate statistical test for each variable was determined based on the distribution of the data, as assessed by the Kolmogorov-Smirnov Z-test. Continuous variables were analyzed using either independent *t*-tests or Mann-Whitney *U*-tests, while categorical variables were evaluated using chi-square and Fisher exact tests. Multivariate linear regression models were employed to ascertain the independent risk factors associated with prolonged length of stay (LOS). The independent risk factors for the selection of surgical treatment were determined using a multivariate logistic regression analysis, with a *p*-value of less than 0.05 considered to be statistically significant.The analysis uses a Generalized Additive Model (GAM) to examine the relationship between LOS and ISS.

## Results

### Patient characteristics

From 2020 to 2024, a total of 70 blunt splenic injuries were admitted to our hospital. A total of 52 patients with BSI (65.4% male) were included in the study, with a mean age of 6.2 ± 2.8 years, a mean length of hospitalization of (14.0 ± 9.7) days, and a mean World Society of Emergency Surgery (WSES) classification of grade 2. The most common mechanism of trauma was motor vehicle accidents (34.6%). The additional patient demographics for the entire cohort are presented in Table 1.

**Table 1:** Characteristics of the study population.

Total study population (*N* = 52)	Mean + SD
Age	6.2 ± 2.8
BMI (kg/m^2^)	16.2 ± 2.7
Pulse rate	113.8 ± 24.2
Systolic blood pressure (mmHg)	106.0 ± 15.3
Hb (mmol/L)	101.5 ± 19.3
Total length of stay	14.0 ± 9.7
Body temperature (℃)	36.7 ± 0.5
Length of stay in ICU	2.6 ± 4.5
ISS	19.5 ± 14.9
Gender	*N* (%)
Male	34 (65.4%)
Female	18 (34.6%)
Trauma mechanism	
Car accident injuries (motor vehicle)	18 (34.6%)
Car accident injuries (non-motorized)	4 (7.7%)
Low fall injuries (<1 m)	8 (15.4%)
Fall injuries (>1 m)	11 (21.2%)
Bump on the ground	8 (15.4%)
A direct hit to the abdomen	3 (5.8%)
Splenic injury grade	
Grade Ⅰ	5 (9.6%)
Grade Ⅱ	15 (28.8%)
Grade Ⅲ	18 (34.6%)
Grade Ⅳ	6 (11.5%)
Grade Ⅴ	8 (15.4%)
WSES classification	
Grade Ⅰ	17 (32.7%)
Grade Ⅱ	14 (26.9%)
Grade Ⅲ	10 (19.2%)
Grade Ⅳ	11 (21.2%)
Isolated splenic injury	16 (30.8%)
Combined abdominal symptoms	7 (13.5%)

CT, computerized tomography; ISS, Injury Severity Score; SD, standard deviation, WSES, World Society of Emergency Surgery, Hb, hemoglobin.

### Injury characteristics

A total of 69.2% of patients were polytraumatized, exhibiting higher Injury Severity Scores (ISS) (6.2 ± 3.1 vs. 25.4 ± 14.2, *p* < 0.001) and accelerated heart rates (99.5 ± 12.9 vs. 120.0 ± 25.4, *p* = 0.004) compared to non-polytraumatized patients. The mean hemoglobin (Hb) concentration on admission in BSI patients was 101.5 ± 19.3 mmol/L, with a mean ISS of 19.5 ± 14.9. A total of 3% of patients exhibited hemodynamic instability, with a significantly higher Injury Severity Score (ISS) in the stable group compared to the unstable group (17.7 ± 13.2 vs. 28.4 ± 19.4, *p* < 0.05). Additionally, the stable group demonstrated a higher temperature than the unstable group (36.8 ± 0.4 vs. 36.4 ± 0.5, *p* < 0.05). These patients were also more prone to manifestations of shock, which led to a higher rate of blood transfusion (20.9% vs. 77.8%; *p* < 0.001). The mean splenic injury grade on admission was 3.0, with 26.9% of patients exhibiting a higher injury grade (grades IV-V) as determined by CT imaging.

**Figure 2:**
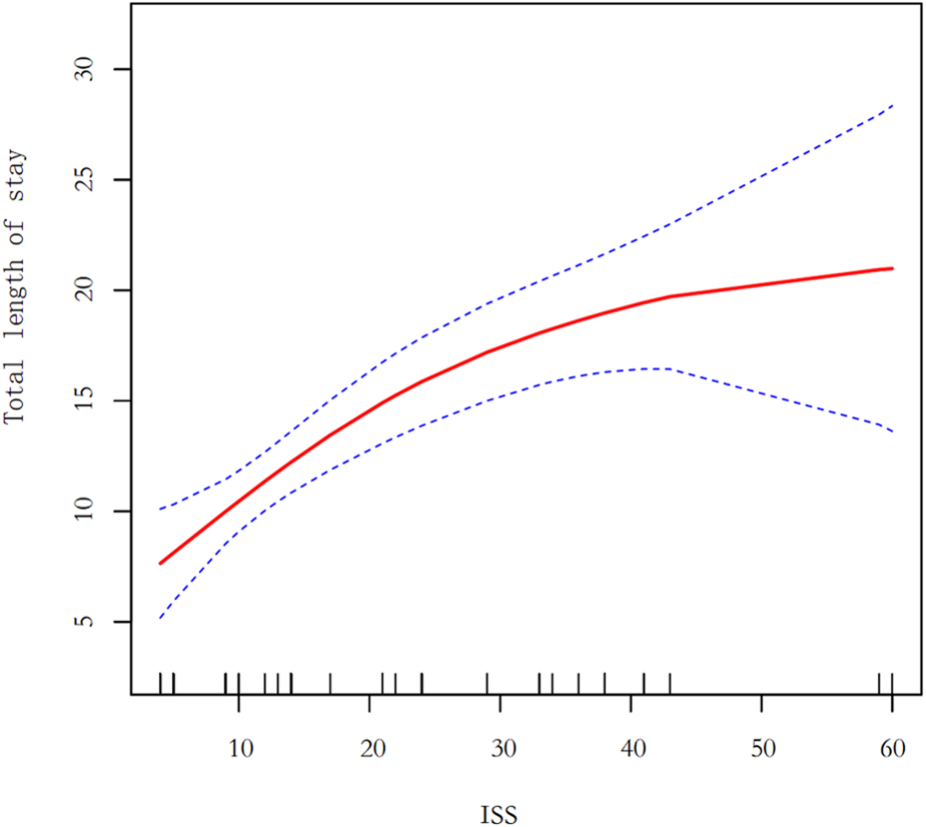
Generalized additive model. ISS, injury severity score.

As can be seen in the Figure 2, the smoothed curve shows a gradual upward trend in hospital length of stay at lower ISS values, suggesting a corresponding increase in hospital length of stay as injury severity increases. In the medium to high ISS range (e.g., 40–60), the smoothed curve tends to flatten or even decrease slightly, possibly reflecting the lack of significant further prolongation of hospital time in patients with high ISS, suggesting that there may be other influences, such as the effects of more intensive care or increased mortality.

### Operative management

The results are presented in tabular form. The mean and standard deviation (SD) were calculated for each variable. The Kruskal-Wallis rank sum test was used for continuous variables, while the Fisher exact probability test was employed for count variables with a theoretical number of less than 10.

### Management

A total of 88.5% of patients underwent non-operative management (NOM), with 11% of these cases involving embolization of the spleen for hemodynamic instability and arterial hemorrhage in Table 2. The remaining 11.5% underwent splenectomy or partial splenectomy for indications requiring surgical exploration, including comorbid gastrointestinal perforation, or peritonitis. The majority of patients who underwent surgical intervention were selected for this procedure due to the presence of comorbid abdominal symptoms (66.7%). One patient died as a result of brain injury. The surgical group exhibited higher transfusion requirements and ISS scores than the non-surgical group (21.7% vs. 100%, *p* < 0.001; 37.7 ± 16.1 vs. 17.2 ± 13.1, *p* < 0.001, respectively). The results of the multivariate logistic regression analysis indicated that the combination of abdominal symptoms, including gastrointestinal perforation, and peritonitis, was significantly associated with the decision to perform surgery [*β* (95% CI), *P* value/OR (95% CI), *P* value; *p* < 0.05] in Table 3.

**Table 2:** Comparison of characteristics: NOM vs. OM in patients.

Treatment method	NOM	OM	Standardize diff.	*P*-value
*N*	46	6		
Age (years)	6.2 ± 2.9	5.8 ± 1.9	0.2 (−0.7, 1.0)	0.698
BMI (kg/m^2^)	16.3 ± 2.8	15.3 ± 1.5	0.4 (−0.4, 1.3)	0.410
Pulse rate	111.6 ± 22.9	130.5 ± 29.4	0.7 (−0.1, 1.6)	0.071
Systolic blood pressure (mmHg)	105.9 ± 14.7	107.0 ± 20.7	0.1 (−0.8, 0.9)	0.872
Hb (mmol/L)	101.0 ± 20.3	105.8 ± 6.2	0.3 (−0.5, 1.2)	0.566
Total length of stay	13.2 ± 9.1	20.5 ± 12.0	0.7 (−0.2, 1.6)	0.080
Body temperature (℃)	36.7 ± 0.5	36.6 ± 0.5	0.2 (−0.7, 1.0)	0.675
Length of stay in ICU	2.3 ± 4.1	5.0 ± 6.6	0.5 (−0.4, 1.4)	0.160
ISS	17.2 ± 13.1	37.7 ± 16.1	1.4 (0.5, 2.3)	<0.001
Gender			0.5 (−0.4, 1.3)	0.326
Male	29 (63.0%)	5 (83.3%)		
Female	17 (37.0%)	1 (16.7%)		
Trauma mechanism			1.4 (0.5, 2.3)	0.298
Car accident injuries (motor vehicle)	14 (30.4%)	4 (66.7%)		
Car accident injuries (non-motorized)	4 (8.7%)	0 (0.0%)		
Low fall injuries (less than 1 m)	8 (17.4%)	0 (0.0%)		
Fall injuries (>1 m)	10 (21.7%)	1 (16.7%)		
Bump on the ground	8 (17.4%)	0 (0.0%)		
A direct hit to the abdomen	2 (4.3%)	1 (16.7%)		
Transfuse blood	10 (21.7%)	6 (100.0%)	2.7 (1.7, 3.7)	<0.001
Hemodynamics (blood flow)			0.4 (−0.4, 1.3)	0.270
stabilize	39 (84.8%)	4 (66.7%)		
instability	7 (15.2%)	2 (33.3%)		
Isolated splenic injury			0.4 (−0.5, 1.2)	0.426
Yes	15 (32.6%)	1 (16.7%)		
No	31 (67.4%)	5 (83.3%)		
Mortality	1 (2.2%)	0 (0.0%)	0.2 (−0.6, 1.1)	0.715
Combined abdominal symptoms	3 (6.5%)	4 (66.7%)	1.6 (0.7, 2.5)	<0.001
Splenic injury grade			0.6 (−0.3, 1.4)	0.175
Low-grade	35 (76.1%)	3 (50.0%)		
high-grade	11 (23.9%)	3 (50.0%)		
WSES classification			1.2 (0.3, 2.1)	0.086
Minor (WSES class I)	17 (37.0%)	0 (0.0%)		
Moderate (WSES classes II and III)	21 (45.7%)	3 (50.0%)		
Severe (WSES class IV)	8 (17.4%)	3 (50.0%)		

BMI, body mass index; ICU, intensive care unit; LOS, length of stay; SD, standard deviation; ISS, Injury Severity Score; NOM, non-operative management, OM, operative management.

**Table 3:** Multivariate logistic regression analysis.

Exposure	Model 1	Model 2
Combined abdominal symptoms	28.67 (3.65, 225.30) 0.0014	50.81 (3.95, 653.89) 0.0026

The data in the table: OR (95% CI) *P* value Model 1: None; Model 2 adjust for: Age; Gender; BMI (kg/m^2^).

### Treatment outcomes

Twenty-four patients were admitted to the intensive care unit on admission and were on bed rest from the time of admission; patients were not active until discharge. With polytrauma, patients with high ISS scores had a longer hospital stay (all *p* < 0.05).

## Discussion

In managing pediatric closed splenic injuries, clinical judgment and early resuscitation are critical in choosing a management strategy. In patients who are initially hemodynamically unstable, non-surgical management only applies after effective resuscitation and hemodynamic stability have been achieved. This allows the preservation of splenic function and minimization of invasive manipulation while ensuring patient safety. Our results show that some patients can remain stable after resuscitation, resulting in a high success rate of non-surgical management. Surgery should be performed if the patient remains unstable after resuscitation or develops symptoms such as gastrointestinal perforation or peritonitis.

### Comparison with existing literature

In the original APSA 2000 version of the guidelines, it was determined that the grade of injury as defined by the AAST was a significant factor in determining the appropriate hospital disposition for children. Specifically, children with grade I to III injuries were admitted to the general ward, while those with grade IV injuries were admitted to the ICU (8). Some studies have demonstrated that children with uncomplicated grade I to II liver or spleen injuries, in the absence of other significant injuries, can be managed with medical observation and discharged from the emergency department without the need for blood transfusions or other specialized interventions (such as surgery) (9).

In contrast, the WSES guidelines (7) suggest that the treatment plan should not be determined based on the classification of splenic injury. Instead, they advocate for the use of physiological parameters as the primary reference data. The WSES classification system categorizes splenic injuries into three distinct groups: mild, moderate, and severe.

Mild splenic injuries are classified as WSES class I and include hemodynamically stable AAST-OIS class I-II blunt and penetrating lesions. Moderate splenic injuries are classified as WSES class II and include hemodynamically stable AAST-OIS grade III blunt and penetrating lesions. Severe splenic injuries are classified as WSES grade III and include hemodynamically stable AAST-OIS grade IV-V blunt and penetrating lesions. Severe splenic injury: The WSES grade IV encompasses hemodynamically unstable AAST-OIS grades I-V blunt and penetrating lesions. It is recommended that the first non-operative management (NOM) attempt be undertaken in patients who are hemodynamically stable and do not have other abdominal organ injuries that require surgical intervention, irrespective of the grade of injury. In contrast, surgical treatment is indicated in cases of hemodynamic instability or other indications for open surgery, such as peritonitis, injury to cavity organs, extra-intestinal clefts, and impaction.

In a similar analysis of the National Trauma Data Bank (NTDB), Nanee and colleagues (10) examined the timing of surgical interventions for BLSI in children. They found that the failure of non-surgical treatment was based on physiological parameters rather than the anatomical severity of the injury.

This conclusion has also been corroborated by the most recent iteration of the APSA guidelines (2023) (4). The APSA guidelines (2023) additionally advise that patients with BLSI who have undergone transfusion of red blood cells but whose vital signs remain unstable should undergo surgical exploration and control of bleeding. Furthermore, to rule out other abdominal injuries that may have contributed to the instability of the patient, surgical exploration should be performed. Moreover, several studies have indicated that hemodynamic status on admission is not a significant prognostic indicator of NOM failure, and thus should not be regarded as an absolute contraindication to NOM (11–15). Our study also adheres to this perspective. As a result of advancements in medical technology, some of the previous therapeutic strategies have been enhanced. The implementation of effective life support techniques and vascular interventions will contribute to a gradual transition in the therapeutic strategies employed for blunt splenic injuries.

### Clinical significance

Nonoperative management (NOM) is a treatment option that avoids surgical intervention for patients with splenic trauma in the pediatric population. The failure rate for NOM in this patient group is reported to be between 2% and 5% (16, 17). It is noteworthy that evidence suggests that the rate of nonoperative management (NOM) failure is highest at four hours post-admission and then gradually declines over 36 h (16). In general, the majority (72.5%) of cases where NOM is unsuccessful appear to occur within the first week following trauma, with 50% occurring within the first 3–5 days (18). The presence of hemodynamic instability is typically discernible in patients upon admission or within 12 h of the injury (19). Patients with higher Injury Severity Scores (ISS) are more likely to fail nonoperative management (NOM). As evidenced in the literature, the two ISS values significantly associated with NOM failure are greater than 15 (14) or 25 (18). This finding is consistent with the increased risk of associated lesions at higher Injury Severity Scores (ISS). This finding was not reflected in my study data.

NOM appears to be more effective in children than in adults and is therefore more commonly used for pediatric splenic trauma in these patients. This has also been associated with reduced costs and length of hospital stay, reduced need for blood transfusions, vaccinations, and antibiotic treatments, as well as higher immunity and lower infection rates (8, 20–23). The reason for the superior outcomes of NOM in children compared to adults is unclear. However, it is postulated that this may be related to certain unique pediatric characteristics, including a thicker splenic envelope, a higher proportion of myoepithelial cells, and more effective contraction and retraction of small splenic arteries (24–29). Several studies have demonstrated low adherence to APSA guidelines in non-pediatric trauma centers. For further details, please see references (6, 22, 30–34). A higher probability of experiencing non-operative management (NOM) has been demonstrated in pediatric trauma patients treated in specialized centers when compared to those treated in adult trauma centers (22, 30–33). Furthermore, Mooney et al. and Todd et al. have shown that children with splenic injuries are more likely to undergo splenectomy or if treated in an adult trauma center (34, 35).

In children, however, the therapeutic strategy of preserving the spleen is preferred, as the spleen is the largest secondary lymphoid organ, which induces innate and adaptive immune responses against pathogens (36). Splenectomy can result in post-splenectomy-associated menacing infections (OPSI). OPSI is defined as fulminant sepsis, meningitis, or pneumonia caused by Streptococcus pneumoniae (in approximately 50% of cases) (37, 38) and to a lesser extent by Haemophilus influenzae type B and Meningococcus meningitis. OPSI is a medical emergency. The risk of OPSI and associated mortality is highest in the first year following splenectomy, particularly in young children. However, it remains elevated beyond 10 years and may persist throughout life. The incidence of OPSI ranges from 0.5% to 2%, with a mortality rate of 30% to 70%. The majority of deaths occur within the first 24 h. Prompt diagnosis and immediate treatment are essential for reducing mortality rates (37, 39–41). The overall risk of OPSI is higher and the mortality rate is greater in children under five years of age with splenic insufficiency or hypersplenism than in adults (37, 42). The risk in neonates exceeds 30% (39). There is evidence that an embolized spleen (in patients with hypersplenism) may maintain its function, but it is reasonable to assume that it is less effective and that vaccination should continue (43–45). The age of the patient at the time of surgery is directly correlated with the time interval between the onset of sepsis. Younger patients tend to present earlier than those who are older. It has been demonstrated that approximately 50% of all cases of OPSI occur within two years following surgery. However, it is important to note that the risk persists throughout the individual's lifetime (46).

At present, there is a paucity of data from global studies on the treatment and prognosis of pediatric splenic trauma. It is therefore of paramount importance that children are encouraged to opt for non-operative management (NOM) and avoid surgical treatment. This study is aligned with the treatment strategy outlined in the internationally recognized guidelines for non-operative management of traumatic splenic rupture, as proposed by the American Pediatric Surgical Association (APSA) Committee 2023.

### Limitations and future directions

It is important to acknowledge the limitations of this study. Firstly, it should be noted that this study was conducted at a single center, which may limit the generalizability of the results to fully reflect the actual situation in other centers or different populations. Secondly, the sample size was relatively small, which may have weakened the stability of the statistical analysis and increased the risk of the results being influenced by chance. Furthermore, as this study was based on existing clinical data, the completeness and consistency of certain variables may have been compromised, potentially impacting the precision of the analysis. Nevertheless, every effort was made to mitigate the impact of these limitations on the study results through the application of rigorous inclusion criteria and a comprehensive data review. A transparent presentation of these limitations can enhance the credibility of this study and inform the design of future multicenter, large-sample-size prospective studies.

#### Management pathway

**Figure 3:**
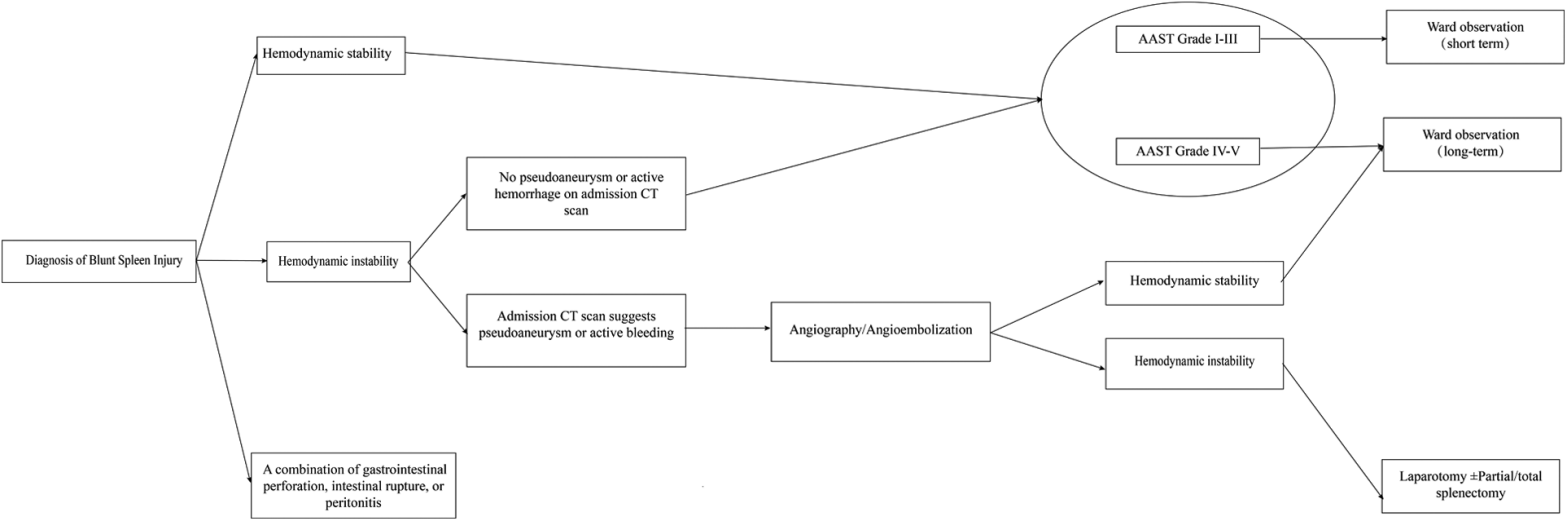
Suggested pathway for management of traumatic blunt splenic injury in children.

Based on the results of our study, a suggested management pathway is outlined that focuses on making a quick decision on which treatment plan to pursue based on the patient's hemodynamic status at the time of admission in Figure 3, with an indication for AG/AE or surgery. The NOM was initially tested in all conditions, and further research is needed to assess the impact of additional injuries and to more accurately predict the length of hospital stay; however, the algorithm can be used as a guide for initial management of the program and may be expanded in the future with further research.

## Conclusion

Our study showed that non-operative management (NOM) is a safe and effective treatment strategy with a high success rate for most children with blunt splenic injury (BSI, especially those who are hemodynamically stable. In addition to reducing surgical complications, non-surgical management reduces children's pain. Surgery should be performed if the patient remains unstable after resuscitation or develops symptoms such as gastrointestinal perforation or peritonitis. Although non-surgical treatment has a high success rate in most patients, minimally invasive techniques such as interventional splenic embolization are effective in reducing the need for splenectomy in selected high-risk cases. Further studies should focus on the long-term efficacy of different treatment strategies and validate the potential of new imaging modalities in conservative management.

## Data Availability

The raw data supporting the conclusions of this article will be made available by the authors, without undue reservation.
